# The effect of anisotropic electrical conductivity of amphiboles on geophysical anomalies observed in subduction zones

**DOI:** 10.1038/s41598-025-98025-9

**Published:** 2025-04-24

**Authors:** Simone Bernardini, Giancarlo Della Ventura, Frank C. Hawthorne, Augusto Marcelli, Francesco Salvini, Boriana Mihailova

**Affiliations:** 1 Fachbereich Erdsystemwissenschaften, Universität Hamburg, Grindelallee 48, 20146 Hamburg, Germany; 2 Dipartimento di Scienze, Università di Roma Tre, Largo S. Leonardo Murialdo 1, 00146 Rome, Italy; 3 INFN-LNF, Via E. Fermi 54, 00044 Frascati, Rome, Italy; 4 Department of Geological Sciences, University of Manitoba, Winnipeg, MB R3T 2N2 Canada; 5 International Centre for Material Science Superstripes, RICMASS, Via dei Sabelli 119A, 00185 Rome, Italy

**Keywords:** High-conductivity layers (HCLs), Fe-rich amphiboles, Anisotropic conductivity, Polarons, Resonance Raman scattering, Subduction zones, Solid Earth sciences, Planetary science

## Abstract

Electrical-conductivity anomalies in subduction zones are believed to be strongly connected with global water cycling, volcanism and seismicity. However, the causal atomic-scale processes related to conductivity of rock-forming minerals in subducting rocks are virtually unknown. Here, in situ simultaneous high-temperature Raman spectroscopy and resistivity measurements on riebeckite as a model Fe-rich amphibole in subduction zones show that (1) electronic small polarons, with high mobility along the **c**-axis of the amphibole structure, activate above 500 K; (2) H^+^ starts diffusing within the crystal above 650 K, although electron transport *via* polaron hopping is still the dominant mechanism of charge transfer; (3) the anisotropy in the conductivity is enhanced with increasing temperature, emphasizing the dominant role of *e*^−^ over H^+^ in causing the high conductivity (above 0.01 S/m) of Fe-rich amphiboles. We show that conductivity data obtained *via* magnetotelluric measurements are best modelled by considering the effect of stress-driven alignment of amphiboles during plate motion. Our results thus link atomic- and Earth-scale conductivity processes, significantly improving our understanding of subduction processes.

##  Introduction

Anomalously high electrical conductivity (σ > 0.01 S/m) in the lithosphere-asthenosphere system is well known yet poorly understood. Values up to 0.1 and 1 S/m have been measured in asthenosphere and back-arc/fore-arc regions, respectively^1–4^. In some localities (e.g., East Pacific Rise), the electrical conductivity of the asthenosphere increases along the direction of plate motion^4,5^. There are two main models concerning the anisotropic conductivity observed in the Earth’s interior: (1) stress-driven alignment of interconnected ion-rich fluids^3–8^, and (2) intrinsic activation of charge carriers with anisotropic mobility in preferentially oriented and mutually aligned rock-forming minerals. Examples of the latter process are provided by H^+^ diffusion in olivine^9^ and electron hopping in talc and serpentinite^10^.

**Fig. 1:**
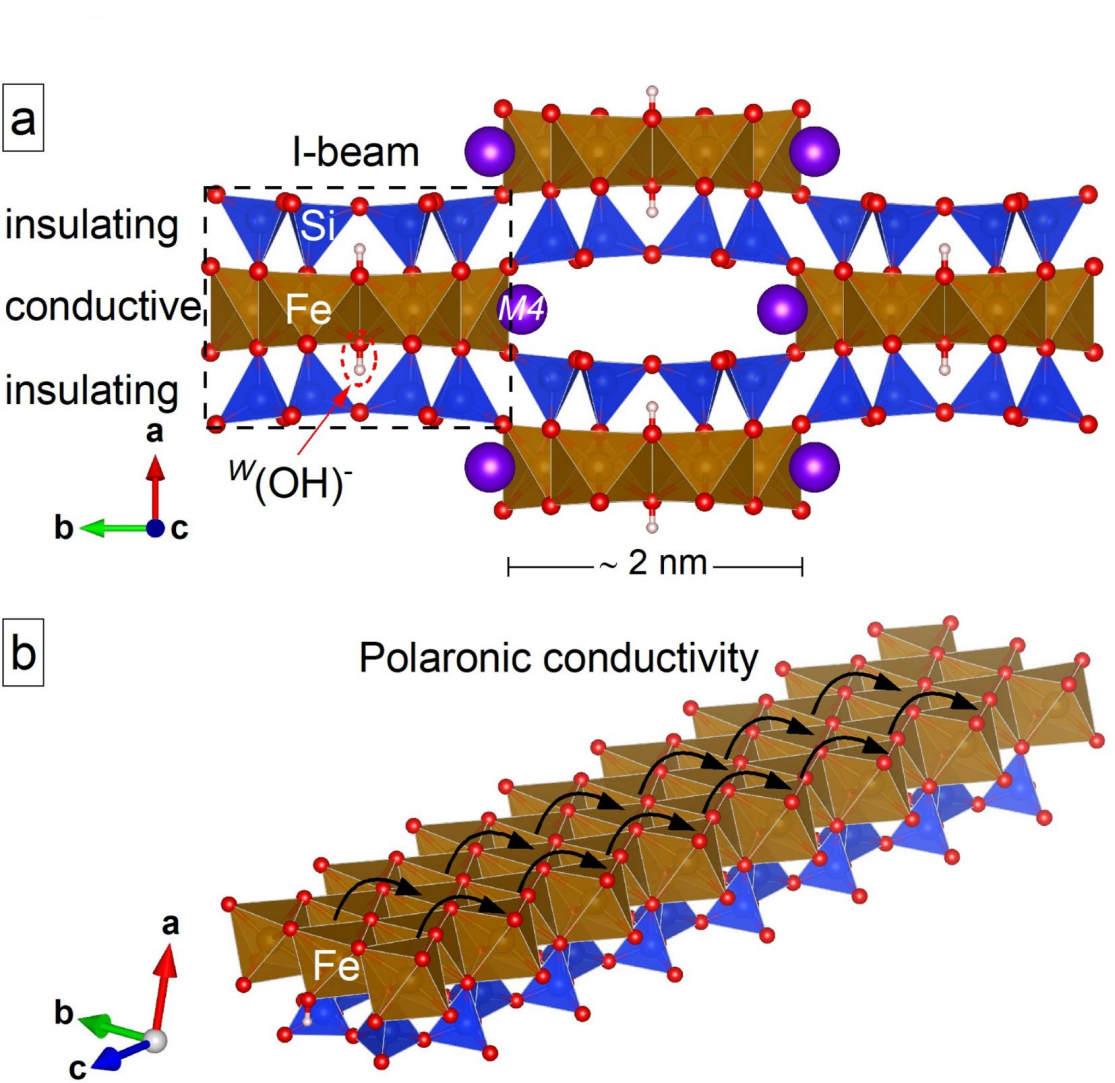
The extremely anisotropic structure of riebeckite. (**a**) View along the c axis of the amphibole structure, which consists of I-beams held together by cations at the *M*(4) site. The I-beam consists of conductive ribbons of edge-sharing FeO_6_ octahedra sandwiched between two insulating silicate ribbons, forming a natural nanorod ( ∼1 × 2 nm^2^ cross section) along **c**. (**b**) A sketch of electronic-polaron hopping (black arrows) along ribbons of FeO_6_ octahedra, resulting in strongly anisotropic conductivity. Atomic structure plotted with Vesta 3.5.8 (https://jp-minerals.org/vesta/en/)^65^.

Amphiboles have gained significant attention in studies of rock conductivity because they are among the primary hydrous phases in the lithosphere, making up as much as 30–40% of continental mid-to-lower crustal rocks and metamorphosed oceanic crust^11^. Furthermore, amphiboles are stable across a broad range of pressures-temperatures^12–14^, and are thus major constituents in rocks spanning a large spectrum of geological conditions from the lower crust to the upper mantle. The composition of these ribbon silicates is extremely variable; their general formula^15^ is written as AB_2_C_5_T_8_O_22_W_2_, where A = Na, K, ☐ (vacancy); B = Na, Ca, Li, Mg, Fe^2+^, Mn^2+^; C = Mg, Fe^2+^, Fe^3+^, Mn^2+^, Al, Ti, Cr, V; T = Si, Al, Ti; and W = OH, F, Cl, O^2−^. Their structure is strongly anisotropic, consisting of ribbons of edge-sharing MO_6_ octahedra sandwiched between electrically insulating ribbons of SiO_4_ tetrahedra to form I-beams (Fig. 1a): natural nanorods aligned parallel to the crystallographic **c** axis (Fig. 1b). This atomic arrangement results in very anisotropic physical properties which strongly affect the behaviour of amphibole-bearing rocks. For example, elastic P-waves propagate faster along the **c** axis than normal to the **c** axis (the direction of amphibole-crystal elongation); thus stress-driven crystal preferred orientation (CPO) of amphiboles is assumed to be the main source of the seismic anisotropy observed worldwide in the deep crust, subducting slab and mantle-wedge regions^16–21^.

Experiments on Fe-bearing amphiboles and amphibole-bearing rocks show high electrical conductivity at high temperature^22–26^. Moreover, the conductivity along the I-beams (i.e., along the **c** axis, see Fig. 1b), σ_||_, is about five to six times larger than the conductivity normal to **c** (σ_⊥_)^24,27^, in agreement with the findings of Zhou et al.^28^ which show that at 1.0 GPa, amphibole-containing rocks have strong anisotropy of both wave (*V*_p_ and *V*_s_) velocity and electrical conductivity, the latter increasing with rising temperature (from 11.1% at 523 K to 25.2% at 973 K). The stress-driven CPO of Fe-amphiboles is thus expected to contribute to both electrical and seismic anisotropy within subducting oceanic crusts, as is the case for other hydrous minerals such as talc and serpentine^10^.

Based on the low activation energy (< 1 eV, see Table 1), the conductivity of amphiboles has been assumed for decades to be due to Fe-related small electronic polarons, i.e., hopping electrons coupled with longitudinal optical (LO) phonons^22,24,25,27,29^. This inference is consistent with the low mobility of H^+^ within the amphibole structure^30^. Wang et al.^23^ showed that the enhanced electrical conductivity of amphibolites for temperatures above 800 K (at 0.5 GPa) is due to Fe²⁺ ↔ Fe³⁺ charge transfer, rather than the production of aqueous fluids. Similarly, Della Ventura et al.^26^ observed a rise of electrical conductivity in a single crystal of riebeckite due to Fe^2+^ → Fe^3+^ + *e*^*−*^ exchange induced by thermal treatment (above 500 K). The activation of Fe-related electron hopping in amphiboles leads to high conductivity (up to 10^−^² to 10^−^¹ S/m) at relatively modest temperatures without the need to invoke partial melting or the involvement of aqueous fluids^26,30^. A notable exception is tremolite (Table 1). The activation energy for conductivity in this Fe-poor amphibole is 3.79 eV, suggesting a different conduction mechanism such as ion (Mg²⁺, Ca²⁺) diffusion^29,31^.

**Table 1:** Activation energy *E*_*a*_ for single-crystal amphiboles, covering a broad range of Fe/(Fe + Mg) ratios, as reported in the literature.

Method	Sample identification	Fe/(Fe + Mg)	E_a_ (eV)	References
Impedance spectroscopy	arfvedsonite	~ 1	0.4 (σ_||_)	Schmidbauer et al.^24^
	edenitic-hornblende	0.75	0.58 (σ_||_)	Schmidbauer et al.^25^
	edenite	0.36	0.70–0.80	Hu et al.^22^
	kaersutite	0.25	0.61 (σ_||_)	Schmidbauer et al.^25^
	Mg-hornblende	0.22	0.73–1.06 (σ_||_)	Schmidbauer et al.^25^
	tremolite	0.01	3.79	Shen et al.^31^
dc electrical resistance	riebeckite (crocidolite)	~ 1*	0.69 (σ_||_)	Littler and Williams^43^
	riebeckite	~ 1*	0.9 (σ_||_)	Parkhomenko^45^
	riebeckite	0.95	0.77 (σ_||_)	Della Ventura et al.^26^
	riebeckite	0.95	0.53 (σ_||_) (300–720 K)	This work
	not given	0.19	0.54(σ_||_) and 0.57(σ_⊥_**)**	Tolland^27^

The corresponding conductivity data are given in Fig. 4. An asterisk indicates an assumed value (not shown in Fig. 4).

Despite the paramount implications for planetary-scale phenomena (e.g., high conductivity, water cycling, earthquake activity, arc magmatism, and ore-forming processes), the formation of thermally activated FeO₆-related polarons with high mobility along the I-beam (Fig. 1b) has only recently been proven directly in several amphibole species^26,32–36^. This was done using the direction-dependent appearance of resonance Raman scattering (RRS) at elevated temperatures^32–36^ and in situ simultaneous measurement of electrical resistivity and Fe oxidation state^26^. Polarons arise from the coupling between conduction electrons and longitudinal optical (LO) polar phonons, carrying local structural distortions due to Fe²⁺ ↔ Fe³⁺ charge transfer. Resonance Raman scattering is widely used in solid-state physics to obtain detailed information on specific electronic states and elementary excitations involved in transport phenomena^37^. In dielectric materials, RRS occurs *via* Fröhlich interactions^38^, i.e., electrostatic interactions between electrons and phonons. Under resonance conditions, a photon from the laser beam interacts with both the phonon and the electron, altering the symmetry selection rules. As a result, the polar optical modes associated with the atomic species involved in the electron transition (specifically the Fe–O modes in our case) are enhanced^39^. In centrosymmetric crystals such as amphiboles, this leads to distinct spectral changes: normal Raman-active (nonpolar) modes are suppressed while infrared-active modes are enhanced. RRS thus provides direct and robust evidence for the activation of small electronic polarons^32–36^. The temperatures of beginning and completion of polaron activation (T’ and T’’, respectively), the average magnitude of polaron dipoles, and the degree of polaron-dipole alignment may vary with amphibole composition^33^. For example, a high Fe^2+^/Mg ratio in the ribbons of MO_6_ octahedra or a filled *A* site promote the activation of polaron hopping^32–36^. However, the appearance of RRS in actinolite^34^ has shown that even a minor amount of Fe^2+^ in magnesian amphiboles is sufficient to trigger polaron hopping, albeit at significantly higher temperatures compared to Fe^2+^-rich amphiboles^33^. Riebeckite (^A^☐^B^Na_2_^C^((Fe^2+^> Mg)_3_(Fe^3+^> Al)_2_)Si_8_O_22_(OH)_2_) is an Fe-rich analogue of glaucophane (^A^☐^B^Na_2_^C^((Mg ≥ Fe^2+^)_3_(Al ≥ Fe^3+^)_2_)Si_8_O_22_(OH)_2_), a common amphibole in blueschist-facies rocks associated with subducted oceanic crust^40,41^. In riebeckite, T’ and T’’ match the temperatures at which high-conductivity layers (HCLs) are observed in warm and cold subduction environments^35^. High-temperature polarized Raman spectroscopy also revealed the activation of a second type of charge carrier near T’’: delocalized H^+^ ions^32,34–36,42^. However, Raman spectroscopy alone cannot quantify the contribution of the different types of charge carriers to the overall conductivity.

Here we report our results from in situ high-temperature polarized Raman spectroscopy coupled with resistivity measurements on riebeckite up to ~ 720 K, a temperature above which irreversible Fe oxidation and dehydrogenation take place in the presence of O_2_^42^. This approach allowed us to monitor the development of mobile charge carriers and simultaneously to quantify the evolving electrical conductivity along (σ_||_) or perpendicular (σ_⊥_) to the I-beams of the structure. The measured σ_||_ and σ_⊥_ values were then used to address the effect of stress-driven alignment of amphiboles on the electrical structure of subducting plates, providing a key to improve the interpretation and modelling of Earth-scale conductivity data obtained *via* magnetotelluric (MT) measurements.

## Results

**Fig. 2:**
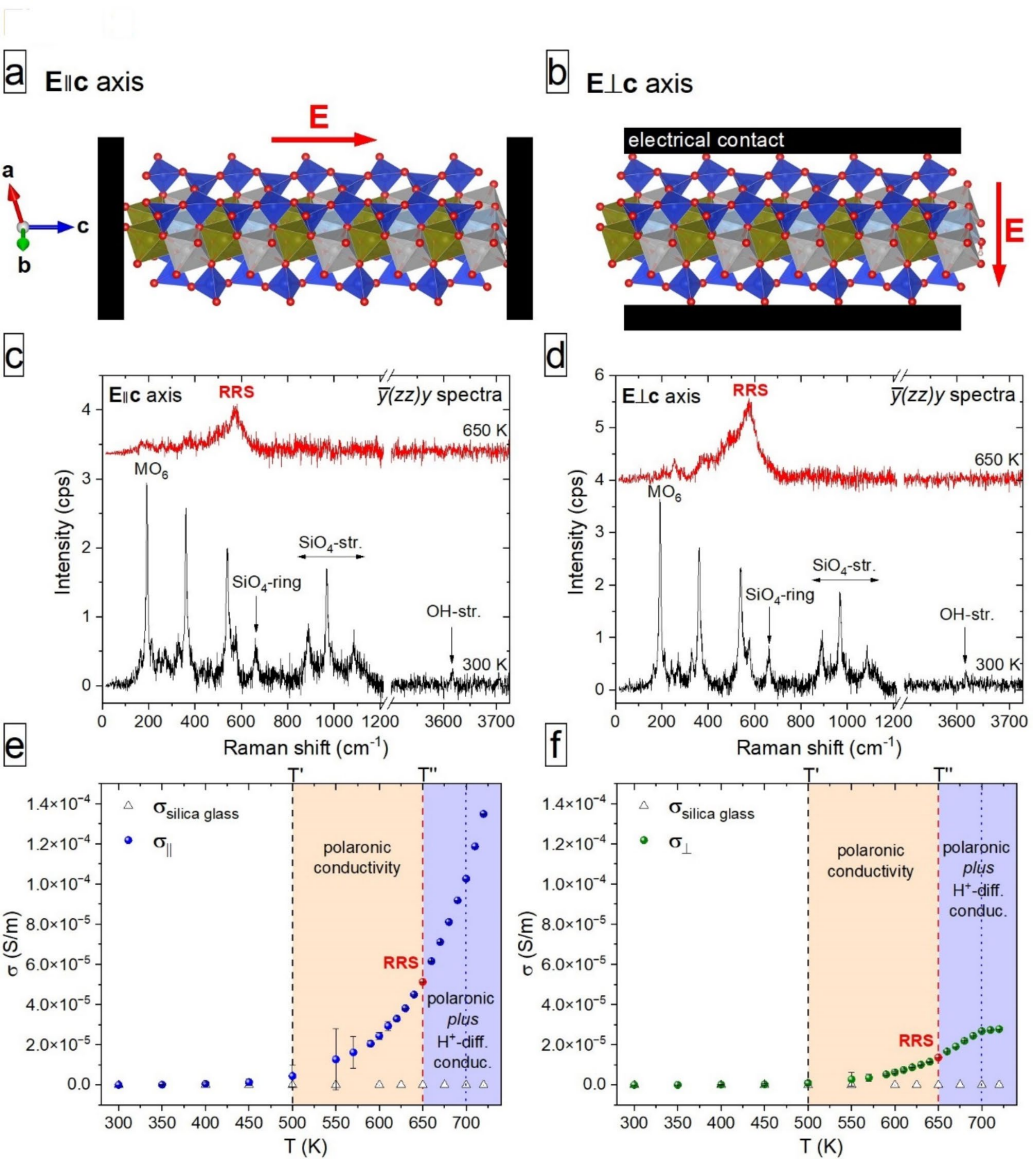
Atomistic interpretation of the electrical conductivity of riebeckite. A sketch of the experimental set-up to measure σ_||_ (a) and σ_⊥_ (b); **E** is the applied dc electric field; in situ temperature-dependent $\:\stackrel{-}{y}\left(zz\right)y$ polarized Raman spectra of riebeckite measured at 300 K and T’’= 650 K with **E**||**c** axis (c) and **E**⊥**c** axis (d); temperature evolution of conductivity measured with **E**||**c**, σ_||_ (e) and with **E**⊥**c**, σ_⊥_, (f); white triangles are *σ*(T) of silica glass as a reference insulator to mark the zero level. The activation energy (in eV) derived from data between T’ and T’’ (shaded in yellow) and above T’’, (shaded in cyan) are also indicated in the plots; the blue dotted lines in (e) and (f) mark the temperature above which σ_⊥_ stops increasing. Atomic structure plotted with Vesta 3.5.8 (https://jp-minerals.org/vesta/en/)^65^.

In situ temperature-dependent parallel-polarized $\:\stackrel{-}{y}\left(zz\right)y$ Raman spectra (Porto’s notation, see experimental methods) collected with an applied dc electric field parallel (**E**_||_) or perpendicular (**E**_⊥_) to the I-beam (see schematic Fig. 2a and b) are shown in Fig. 2c and d, respectively. For Fe-bearing amphiboles, this Raman-scattering geometry provides the temperature of onset of polaron activation (T’) *via* the minimum in the temperature dependence of the wavenumber of the SiO_4_ ring mode at 665 cm^− 1^ and the temperature of cessation of polaron activation (T’’) *via* the appearance of RRS conditions at which all SiO_4_-, Fe^2+^O_6_- and OH-related peaks disappear and a strong scattering arising from LO polar Fe^3+^O_6_ phonon modes appears at ~ 565 cm^− 1^, see ref^32,33,35,36^. For riebeckite, T’ = 500 K and T’’ = 650 K^33,35^. As shown in Fig. 2c and d, temperature-activated RRS occurs exactly at T’’ = 650 K, independent of the direction of the applied external electric field. This evidence provides direct and robust proof for the complete activation of polarons, with dipoles aligned along the amphibole I-beam^32,33,35,36^.

The temperature evolution of σ_||_ and σ_⊥_ is shown in Fig. 2e and f, respectively. Within uncertainties, σ_||_ begins to deviate from the zero level (*σ*(T) of silica glass as a reference insulator, white triangles in Fig. 2e and f) at T’ = 500 K, which is the onset of polaron activation^33,35^ and increases rapidly with increasing temperature. The conductivity in the direction perpendicular to the I-beams (σ_⊥_) also increases above T’, but at a considerably lower rate (note that the scales in both panels are the same). The difference between σ_||_ and σ_⊥_ becomes even larger above T’’= 650 K, which is the temperature of complete polaron activation accompanied by delocalization of H^+ 35,42^. For T > 700 K, σ_||_ continues to increase, whereas σ_⊥_ flattens out (Fig. 2e and f).

**Fig. 3:**
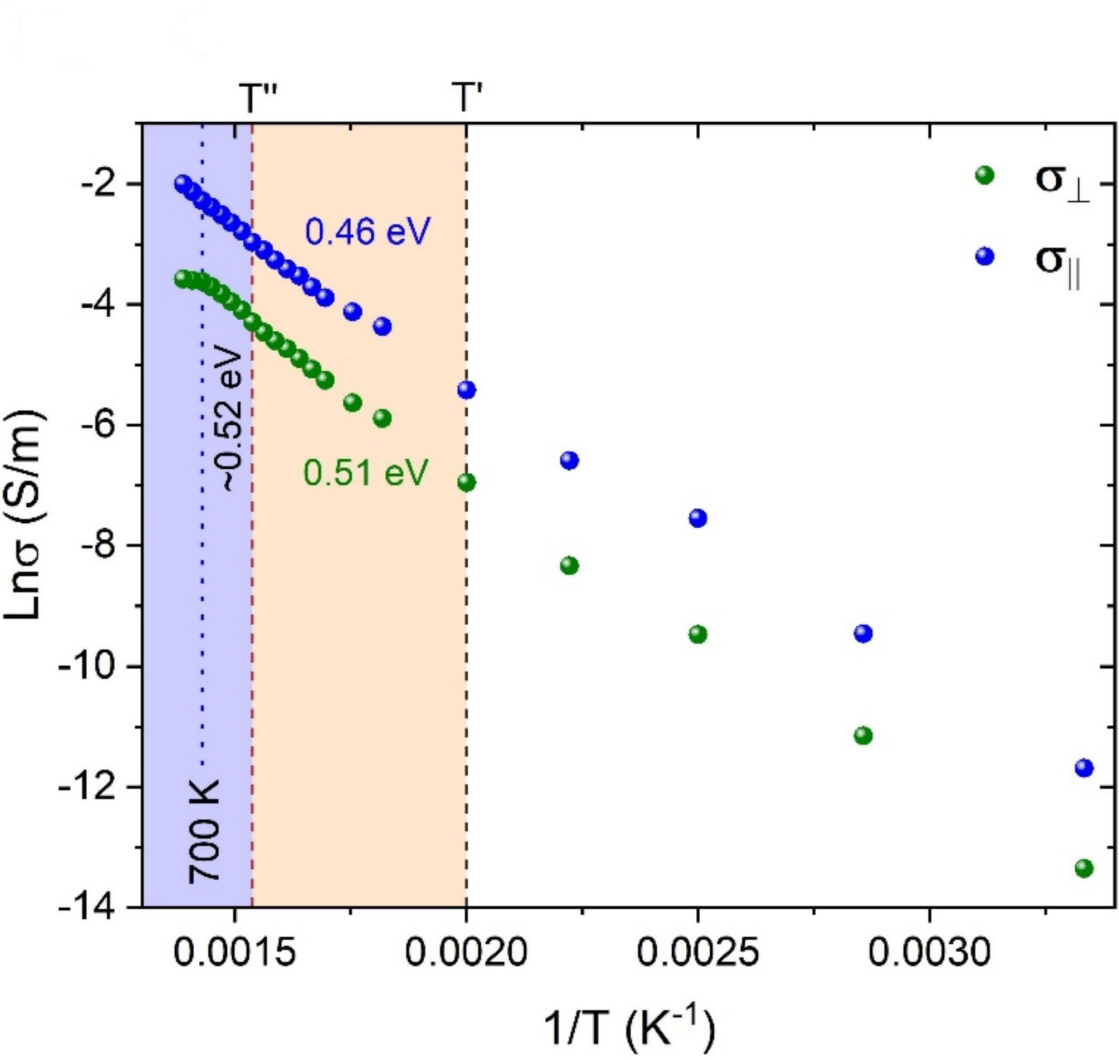
Activation energies for the electrical conductivity of riebeckite. Conductivity (σ_||_ and σ_⊥_) data as a function of temperature plotted in Arrhenius space. The values for the activation energy for “pure” polaronic (T’,T’’) and polaronic *plus* H^+^-diffusion conductivity (T’’,700 K) are indicated. The activation energy derived from σ_||_(T) data in the entire temperature range (300–720 K) is 0.42 ± 0.01 eV. Error bars are smaller than the symbol size.

The activation energies for the conductivity were calculated by applying the Arrhenius equation σ = σ_0_$\:{e}^{-\frac{{E}_{a}}{{k}_{\text{B}}T}}$ (where σ is the conductivity in S.m^− 1^, *k*_B_ is the Boltzmann constant = 8.314 × 10^− 3^ kJ.mol^− 1^K^− 1^, T is the temperature in K, and *E*_*a*_ is the activation energy in kJ.mol^− 1^) to σ_||_(T) and σ_⊥_(T) within two temperature ranges: (1) T’ ≤ T_1_ ≤ T’’, and (2) T’’ ≤ T_2_ ≤ 700 K (see Fig. 3). The conductivity can be thus described as: σ = σ_1_$\:{e}^{-\frac{{E}_{a1}}{{k}_{\text{B}}T1}}$ + σ_2_$\:{e}^{-\frac{{E}_{a2}}{{k}_{\text{B}}T2}}$ where *E*_*a1*_ and *E*_*a2*_ are the activation energies for each temperature range. The values derived from σ_||_(T) and σ_⊥_(T) in the (T’,T’’) range are 44.5 ± 1.8 kJ.mol^− 1^ (0.46 ± 0.02 eV) and 49.6 ± 2.1 kJ.mol^− 1^ (0.51 ± 0.02 eV), respectively (Fig. 3). The activation energies in the range between T’’ and 700 K for both experimental configurations are the same within uncertainties: 51.2 ± 1.1 kJ/mol (0.53 ± 0.01 eV) for σ_||_(T) and 48.6 ± 1.7 kJ/mol (0.50 ± 0.02 eV) for σ_⊥_(T) (Fig. 3). The activation energy derived from σ_||_(T) data in the entire temperature range (300–720 K) is 51.2 ± 0.6 kJ/mol (0.53 ± 0.01 eV).

## Discussion

### Atomic-scale phenomena in riebeckite as a model Fe-amphibole at high temperatures

**Fig. 4:**
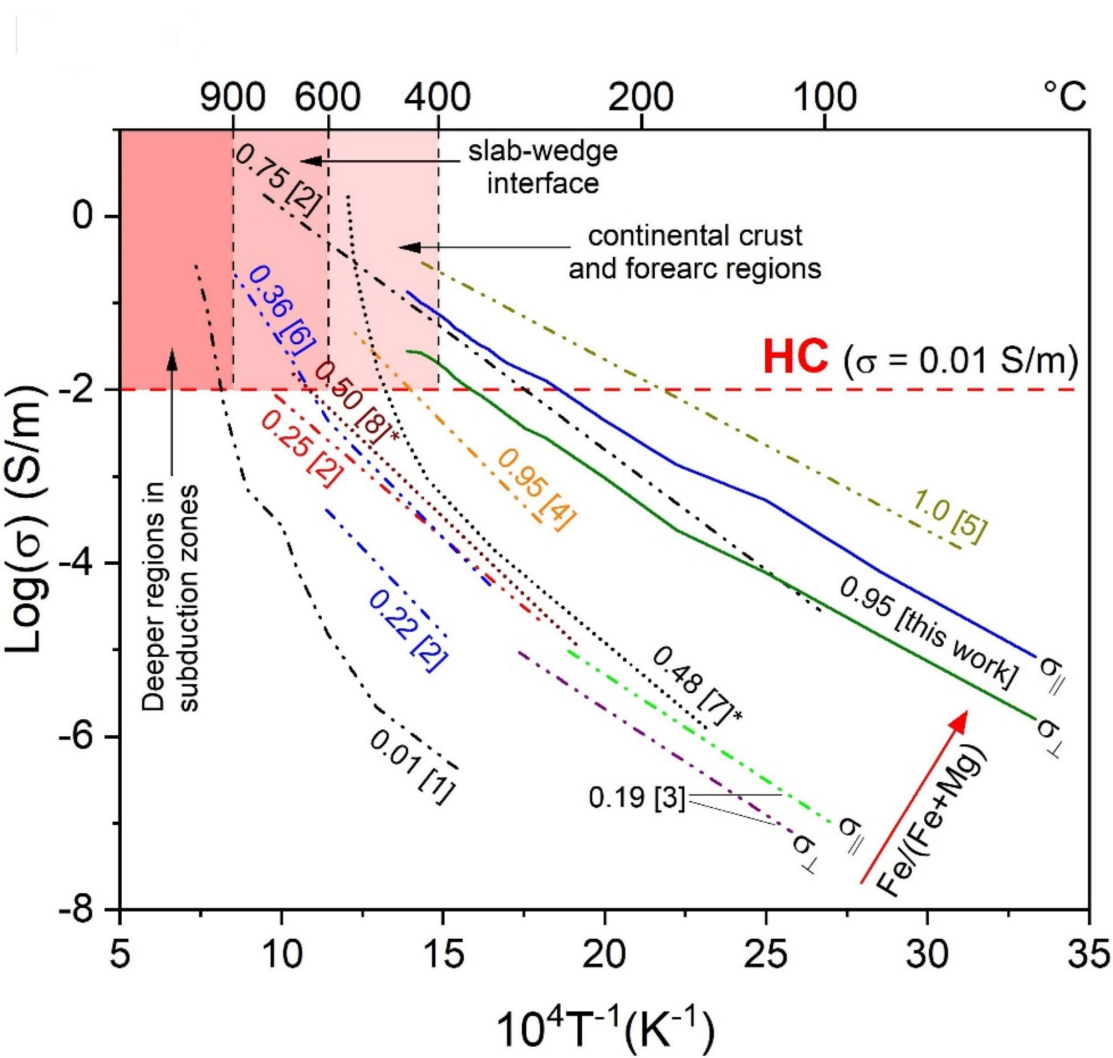
Comparison of electrical conductivity of single crystals of amphibole and amphibolites with anomalous high conductivity (HC) in continental crust/forearc, slab–wedge interface, and deeper regions in subduction zones. Conductivity data for single crystals of amphibole from this work (blue and green solid lines) and from Shen et al.^31^ [1], Schmidbauer et al.^25^ [2], Tolland^27^ (σ_||_ and σ_⊥_) [3], Della Ventura et al.^26^ [4], Schmidbauer et al.^24^ [5], Hu et al.^22^ [6]. Conductivity data for amphibole-bearing rocks (*) from Wang et al.^23^ [7] and Zhou et al.^28^ [8]. The Fe/(Fe + Mg) ratio is indicated for each data set. Note that electrical conductivity increases with the increase of Fe/(Fe + Mg) ratio (red arrow).

Both σ_||_ and σ_⊥_ begins to deviate from the zero level at T’ = 500 K (Fig. 2e and f), signalling the onset of polaron activation^33,35^. However, σ_||_ increases much faster than σ_⊥_ with increasing temperature and at T’’ = 650 K σ_||_ ~ 3.7σ_⊥_. The increase in anisotropic conductivity of riebeckite with increasing temperature, derived from dc measurements, accords with the findings from impedance spectroscopy on amphibolite by Zhou et al.^28^. Given that below T’’, polarons are the main activated charge carriers^35^, this result clearly shows that polaron conductivity in our model amphibole is predominantly along the **c** axis, consistent with the strong structural anisotropy of the amphibole (Fig. 1). The ratio σ_||_/σ_⊥_ at T’’ is in complete agreement with those available for arfvedsonite, another Fe-rich amphibole^24^, and with the data of Tolland^27^ for an amphibole with Fe^2+^/(Fe^2+^+Mg) = 0.19 (see Fig. 4). Strong direction-dependent conductivity with σ_||_ > σ_⊥_ was also observed for a bundle of crocidolite (riebeckite) fibres by Littler and Williams^43^, though they did not provide specific values for the degree of anisotropy.

Above T’’, where most H^+^ is delocalized^35^, σ_||_ increases steadily up to 720 K (Fig. 2e), whereas σ_⊥_ flattens out at 700 K (Fig. 2f) and at 720 K σ_||_ ~ 4.7σ_⊥_. The constant value of σ_⊥_ above 700 K indicates no further formation of charge carriers with mobility preferentially normal to the I-beams. Note that up to ~ 720 K, delocalization of both *e*^−^ and H^+^ is reversible, and only for T > ~ 720 K and in the presence of external O_2_, H^+^ and *e*^−^ are ejected from the crystal and riebeckite irreversibly oxidizes^35,42^. Therefore, both electronic polarons and diffusing H^+^ cations contribute to the conductivity between T’’ = 650 K and 720 K. However, previous infrared spectroscopic analysis suggests that H^+^ diffusion occurs preferentially perpendicular to the I-beams^44^. Hence, the fact that σ_||_ is much larger than σ_⊥_ supports a model in which polaronic conductivity dominates over conductivity due to H^+^ diffusion. Moreover, density-functional-theory simulations on grunerite (nominally ^A^☐^B^Fe^2+^_2_^C^Fe^2+^_5_Si_8_O_22_(OH)_2_) indicate that the experimentally determined T’’ corresponds (in energy units) to the electron energy gap E_g_, which is determined by hybridized *d*-orbitals of ^C^Fe^2+^ and O *p*-orbitals^32^. Thus, above T’’, Fe-rich amphiboles should behave as semiconductors and the number of intrinsic charge carriers (electrons in the conduction band and holes in the valence band) should increase with temperature, which also emphasizes the minor contribution of mobile H^+^ to the amphibole conductivity.

To gain deeper insight into the individual contribution of the two types of charge carriers, polarons and H^+^, to the conductivity anisotropy, we have calculated the activation energies within two temperature ranges (Fig. 3): (1) between T’ and T’’, where mainly FeO_6_-related electronic polarons are activated, and (2) between T’’ and 700 K, where the polaron activation is complete and mobile H^+^ cations are activated, along with intrinsic charge carriers (electrons and holes) in semiconductors. Thus, the former range is indicative of ‘pure’ polaronic activation energy, whereas the latter gives hints on the H^+^-diffusion activation energy (yellow and blue areas in Fig. 3, respectively).

The “pure” polaronic activation energies derived from σ_||_(T) and σ_⊥_(T) in the (T’,T’’) temperature range are 0.46 ± 0.02 eV and 0.51 ± 0.02 eV, respectively (Fig. 3). Our data align with those measured by Tolland^27^ prior to amphibole dehydrogenation (T < 600 K): 0.54 eV along and 0.57 eV perpendicular to the I-beams (see Fig. 4 and Table 1). The smaller *E*_*a*_ for σ_||_(T) compared to that for σ_⊥_(T) provides additional strong support of higher polaron mobility along the I-beams of the structure (Fig. 1b). The activation energies derived from the temperature range between T’’ and 700 K for both experimental configurations are the same within uncertainties: 0.53 ± 0.01 eV for σ_||_(T) and 0.50 ± 0.02 eV for σ_⊥_(T) (Fig. 3). This confirms that the mobile charge carriers activated in this temperature range, which include delocalized H^+^, contribute equally to both σ_||_ and σ_⊥_. Above 700 K, σ_||_ steadily increases, whereas σ_⊥_ flattens out (Fig. 2e and f), indicating no further formation of charge carriers with mobility normal to the I-beams. Note that the activation energy derived from σ_||_(T) data between 300 K and 720 K (0.53 ± 0.01 eV) is compatible with that obtained on Fe-containing amphibole single crystals *via* complex impedance spectroscopy by Hu et al.^22^ (0.7–0.8 eV) and Schmidbauer et al.^25^ (between 0.48 and 1.06 eV, depending on the Fe content of their samples), and that obtained *via* dc measurements by Littler and Williams^43^ (0.69 eV), Parkhomenko^45^ (0.9 eV), Della Ventura et al.^26^ (0.77 eV) and Tolland^27^ (0.54–0.57 eV) (see Table 1). Our data are also consistent with those obtained *via* complex impedance spectroscopy on amphibole-containing rocks by Wang et al.^23^ (0.62 eV) and Zhou et al.^28^ (0.71–0.75 eV).

### Implications of the anisotropic conductivity of amphiboles at subduction zones conditions

Bernardini et al.^35,36^ show that due to the local charge-buffering provided by delocalization of H^+^ (Fig. 1a), external oxygen (^ex^O_2_) is not required to trigger the hopping of polarons in riebeckite. This conclusion has paramount implications in geology because it implies that polaronic conductivity may occur in blue-schist facies rocks associated with subducting oceanic crust at any depth where T > 500 K, regardless the oxygen fugacity. Results from experimental petrology indicate that amphiboles are stable in the mantle lithosphere up to approximately 4 GPa and 1250 K and that their stability is influenced by both water activity and bulk chemical composition^12,13^. Compositions richer in alkalis tend to stabilize the structure at the highest pressures and temperatures^13^. However, as water activity decreases, the stability field shifts to lower pressures while expanding to higher temperatures^14^. In cold subduction zones with lower thermal gradients, amphiboles remain stable to greater depths than in hotter subduction environments (> 150 *versus* ~ 70 km depths)^46^. As shown by Goddat et al.^47^, small-polaron conduction increases with increasing pressure because of its negative activation volume, stressing the role of electron hopping in subducted amphibole-bearing rocks. For riebeckite, in particular, the conductivity increases by approximately one order of magnitude for a pressure increase of 2 GPa in the temperature interval 473 to 873 K^45^. Hu et al.^22^ showed with impedance spectroscopy measurements on a single crystal of edenite that pressure in the range 0.5–2.0 GPa (equivalent to depths of up to ~ 65 km) has a very weak effect on conductivity compared to temperature. Similarly, Shen et al.^31^ observed the same weak pressure effect on the conductivity of tremolite in the range 1.0–2.0 GPa. Moreover, inspection of all data available in the literature shows that there is a clear increase in conductivity with increase of the Fe^2+^/(Fe^2+^+Mg) ratio in the amphibole (Fig. 4), highlighting the dominant role of FeO_6_-related electron hopping for reaching high conductivities (above 0.01 S/m). In summary, the above discussion shows that the main contribution of amphiboles to the bulk conductivity of rocks at lithosphere conditions (e.g., continental crusts, forearc regions, and slab-wedge interface, see Fig. 4) is related to the strongly anisotropic electron hopping along the structural I-beams (Fig. 1b), which can be triggered even by minor amounts of Fe^34^.

**Fig. 5:**
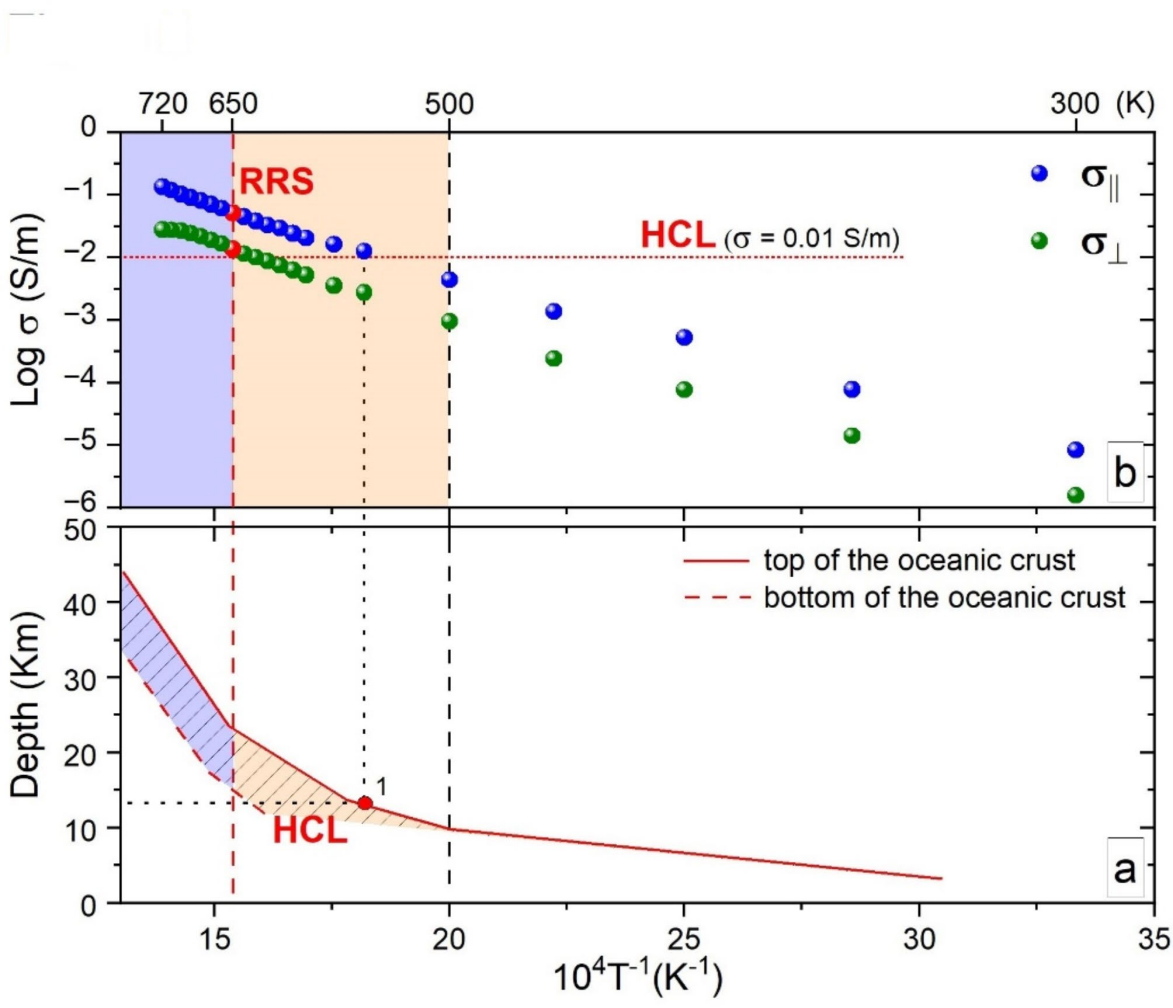
Comparison of the thermal ranges for the activation of polarons as determined by our experiments and the calculated P-T conditions for the oceanic crust subducting beneath SW Japan. Temperature and depth conditions for the oceanic crust subducted beneath SW Japan (modified after Peacock and Wang^46^) (**a**). Temperature evolution of electrical conductivity of riebeckite in different crystallographic directions (σ_||_ and σ_⊥_) (**b**). The yellow shaded areas denote the temperature range of pure polaronic conductivity in our model amphibole, while the cyan areas denote the range of polaronic *plus* H^+^-diffusion conductivity. HCL: high-conductivity layer; RRS: temperature condition for the appearance of resonance Raman scattering in the spectra in Fig. 1; black dashed vertical line (T’=500 K): onset of activation of polarons; red dashed vertical line (T’’=650 K): complete development of polarons and complete delocalization of H^+^ cations.

It is now important to explore the implications of the strongly anisotropic electrical properties of amphiboles for the interpretation of anomalous conductivity measured *via* MT methods worldwide in subduction zones^2^. In the following discussion, we will consider one of the most studied areas in the world: SW Japan. The calculated depth-temperature conditions for the subducting Philippine Sea plate are shown in Fig. 5a and compared with the temperature evolution of σ_||_ and σ_⊥_ of riebeckite (Fig. 5b). According to our experimental results, the development of polaronic conductivity with σ_||_ above 0.01 S/m is expected at the top of the subducting oceanic crust at ~ 15 km depth (point 1 in Fig. 5a). This scenario is in excellent agreement with the electrical conductivity structures observed *via* MT measurements^48–50^.

However, there are two important issues to consider for a proper interpretation of Earth-scale geophysical data: (1) amphiboles subjected to shear stress exhibit strong CPO^16,17^, and (2) MT methods provide primarily the horizontal component of rock conductivity.

**Fig. 6:**
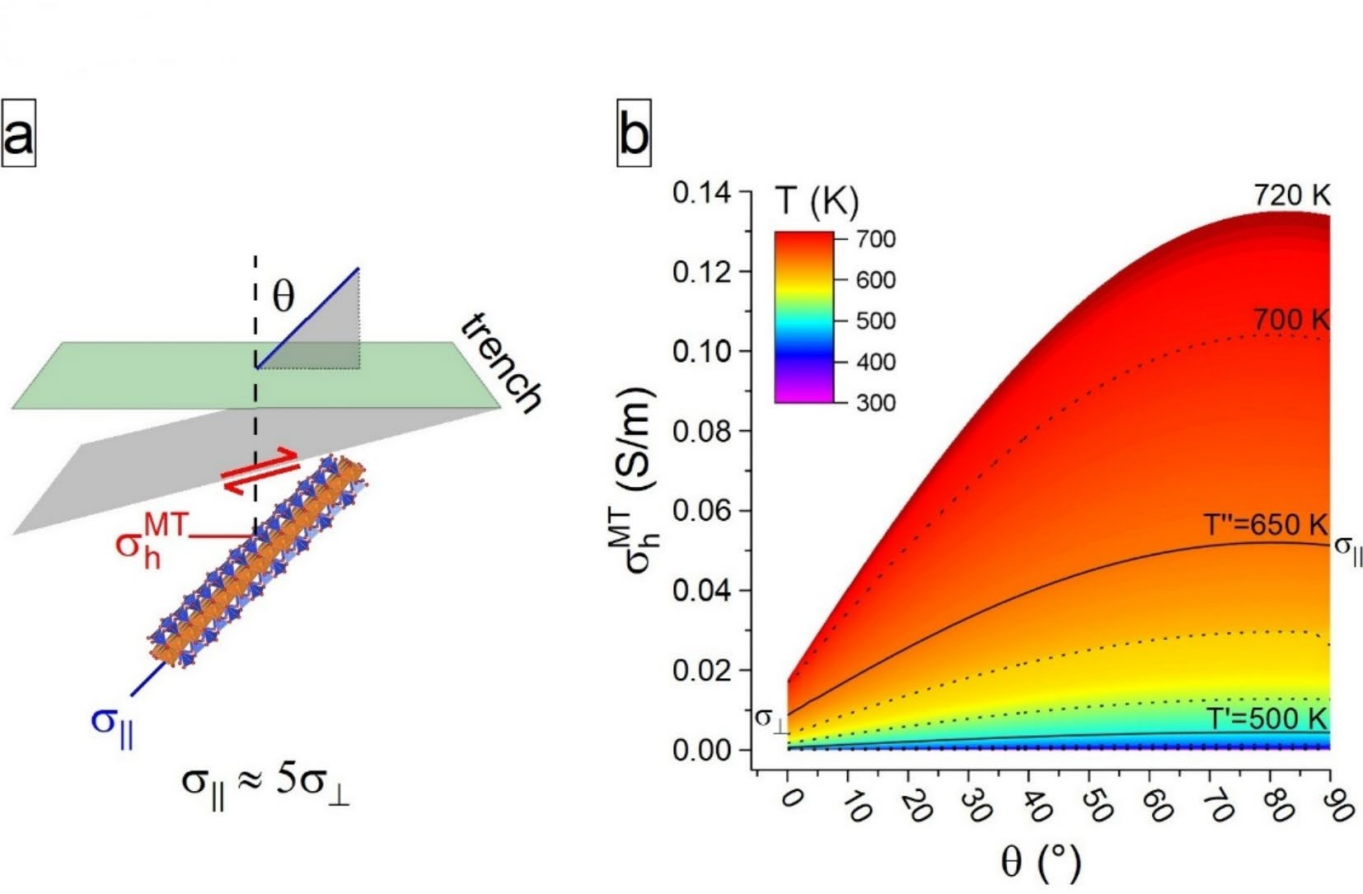
The effect of the CPO on the horizontal component of conductivity in our model sodium amphibole. (**a**) Preferred orientation of σ_||_ of the amphibole in the simple-shear zone; red paired arrows denote the direction of shearing force. (**b**) Isolines of temperature (black lines) showing the relation between $\:{\sigma\:}_{h}^{MT}$ and the *θ* angle between 300 and 720 K. T’: onset of polaron activation. T’’: complete development of polarons.

The CPO of amphiboles depend on both temperature and differential stress^16,17^. The most common CPO in nature, i.e., type-I, with the **c**-axis subparallel to the shear direction and the (100) pole subnormal to the shear plane, develops in the temperature range of activation of anisotropic polarons and under a broad range of stress conditions^16,17^. Anisotropic growth of amphibole grains with this fabric has been also observed in metamorphic rocks from both the Ryoke and Sambagawa belts in SW Japan^51,52^. Hence, for a scenario in which the stress-driven alignment of amphiboles occurs in response to slab descent, the horizontal component can be derived from the in-lab measured σ_||_(T) and σ_⊥_(T) as:

$$\:{\sigma\:}_{h}^{MT}\left(T,\theta\:\right)={\sigma\:}_{\left|\right|}\left(T\right)\text{sin}\left(\theta\:\right)+{\sigma\:}_{\perp\:}\left(T\right)\text{cos}\left(\theta\:\right)$$

where *θ* is the angle between the **c** axis and the direction normal to the Earth’s surface (see Fig. 6a).

The angular dependence of $\:{\sigma\:}_{h}^{MT}\left(T,\theta\:\right)$ at selected temperatures is given in Fig. 6b, which shows that the effect of *θ* on $\:{\sigma\:}_{h}^{MT}$strongly increases with temperature e.g., at 720 K, 0.02 < $\:{\sigma\:}_{h}^{MT}$ < 0.14 S/m for 0° < *θ* < 90°. Hence, for a hypothetical amphibole-bearing subducting slab, $\:{\sigma\:}_{h}^{MT}$ changes because of: (1) increase of temperature along the descending slab, and (2) variation of the θ angle. What is extremely important here is that the CPO of the amphibole (expressed by the θ angle) definitively controls $\:{\sigma\:}_{h}^{MT}$at any given temperature (Fig. 6b). In other words, the evolving stress-driven CPO of the amphibole affects strongly the electrical properties of descending slabs measured *via* MT methods.

**Fig. 7:**
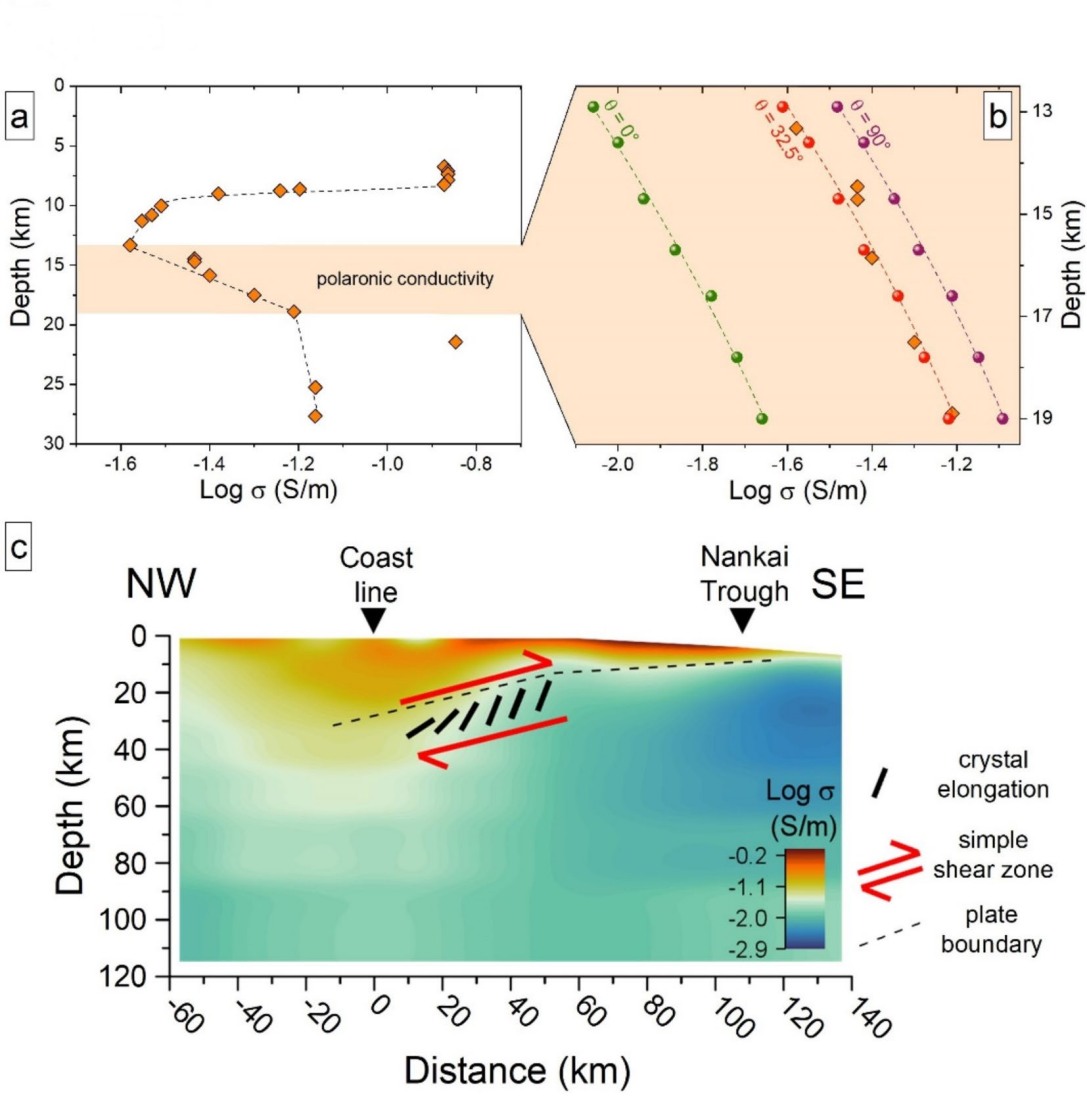
The effect of stress-driven CPO of amphiboles on the electrical structure of a subduction zone. (**a**) Variation of conductivity with depth in SW Japan derived from the transverse MT profile of Kasaya et al.^48^ (see Fig. 7c). The yellow-shaded areas denote the expected depth of development of electronic polarons in our model sodium amphibole (see Fig. 5a). (**b**) Modelling of the conductivity measured in SW Japan (orange diamonds) at the depth of development of polarons. The evolution of $\:{\sigma\:}_{h}^{MT}$ with depth is obtained by integrating the temperature-depth data of Peacock and Wang^46^ (see Fig. 5a) with our in-lab results at different θ values. (**c**) Atomistic interpretation of the regional conductivity model across the Nankai Trough and Kii Peninsula (data from Kasaya et al.^48^). The increase of conductivity between 13 and 19 km (blue to orange area below the plate boundary) closely matches the development of polaronic conductivity in preferentially oriented amphibole crystals during subduction of the Philippine Sea Plate beneath the overlying Eurasian plate.

A plot of the data extrapolated from the 2D MT imaging of Kasaya et al.^48^ shows a steep increase in conductivity along the shear direction of the Philippine Sea Plate subducting beneath SW Japan, between ~ 13 and 19 km depth (yellow shaded area in Fig. 7a). At this depth, the subducted crustal rocks pass through the greenschist metamorphic facies^46^ dominated by amphibole, chlorite, epidote, plagioclase and quartz^53^. It is worth noting that at the temperature conditions of this HCL (see Fig. 5a), the conductivity of our model amphibole is several orders of magnitude greater than that of chlorite (~ 10^− 5^ S/m)^54^, epidote (~ 10^− 3^ S/m)^55^, plagioclase (< 10^− 8^ S/m)^56^, and quartz (~ 10^− 5^ S/m)^57^. This suggests that amphiboles have a more dominant role than other rock-forming minerals in affecting the electrical properties of the descending slab. We note also that the amphiboles constituting the rocks from the Sambagawa belt in central Shikoku (SW Japan) are hornblendes with high Fe content (Fe^2+^ and Fe^3+^ ~ 1.5 and 0.6 apfu, respectively)^58^.

We can use the evolution of $\:{\sigma\:}_{h}^{MT}\left(T,\theta\:\right)$ of our model Fe-amphibole to simulate the sharp increase in conductivity observed *via* MT (yellow shaded area in Fig. 7a). However, it must be emphasized that $\:{\sigma\:}_{h}^{MT}\left(T,\theta\:\right)$ is different for amphiboles of different composition, particularly with different Fe/Mg ratio and *A*-site population^32–36^. Moreover, the slab is a polymineralic system in which several rock-forming minerals affect its electrical properties. Therefore, the following discussion must be considered as a simplified model directed toward increasing our understanding of more complex systems.

The evolution of $\:{\sigma\:}_{h}^{MT}\left(T,\theta\:\right)$ for our amphibole at depths of 13–19 km (calculated using the variation of temperature with depth in Fig. 5a) is given in Fig. 7b and compared with the MT data from Kasaya et al.^48^ (orange diamonds in Fig. 7b). The $\:{\sigma\:}_{h}^{MT}\left(T,\theta\:\right)$ has been calculated at *θ* values between two extreme situations, i.e., *θ* = 0° (assuming the amphibole crystals are oriented vertically during subduction) and *θ* = 90° (assuming the amphiboles are oriented horizontally). Both situations are clearly unrealistic and there is no fit between the experimental data and the simulated data (Fig. 7b). On the other hand, Fig. 7b shows that $\:{\sigma\:}_{h}^{MT}\left(T,\theta\:\:=32.5^\circ\:\right)$ matches very well the MT profile from Kasaya et al.^48^. We need to consider here that in a rock subjected to a simple-shear-stress field (see schematic grey plane in Fig. 6a), the amphibole crystals align progressively with their **a** and **c** axes parallel to the minimum and maximum stress vector, respectively^16,17,51,52^. This being the case, only the *xx* and *zz* components of the conductivity 2nd -rank tensor **σ** will contribute to $\:{\sigma\:}_{h}^{MT}\left(T,\theta\:\right)$. The in-lab measured σ_||_ corresponds exactly to σ_*zz*_, whereas σ_⊥_ is dominated by σ_*xx*_ but contains a small contribution from σ_*yy*_ due to the inclination of σ_⊥_ to the reciprocal-space axis a*. To estimate the influence of this issue on our results, we have averaged σ_⊥_ over an angle *ϕ* ∈ [0, π/2], which represents the extreme situation of randomly oriented **b** and a* axes around the [001] direction. In this case, the best match between $\:{\sigma\:}_{h}^{MT}\left(T,\theta\:\right)$ and the MT profile is obtained for $\:\theta\:\:=37.5^\circ\:$, a value that is very similar (within uncertainties) to that obtained with the formula given above, supporting the validity of our assumption.

Basic structural-geology arguments show that the shallow part of the slab is dominated by simple-shear deformation^59^. Under such conditions, minerals with strong morphological anisotropy (such as amphiboles) grow aligned with their prismatic elongation (|| the **c** crystallographic axis) oriented at ~ 45° to the direction of shearing force^60^. According to our simulation, the dipping angle of the slab should thus be 45°-32.5° = 7.5° (or 45°-37.5° = 12.5° if the angle average of σ_⊥_ is used), which is in close agreement with the value of 7–11° given by Nakanishi et al.^61^. Our simulation is also consistent with the pervasive stress-driven alignment of amphiboles (type-I CPO) observed in the metamorphic rocks from SW Japan^51,52^. Therefore, the stress-driven CPO of the amphibole elegantly explains the increase in conductivity observed between 13 and 19 km beneath SW Japan (blue-to-orange area below the plate boundary in Fig. 7c). This consideration thus implies that preferential orientation of amphibole crystals provides a strongly anisotropic contribution to the bulk electrical conductivity of subducted crustal rocks, with higher conductivity expected along the direction of plate motion.

## Conclusions

This study shows that (1) the onset of activation of FeO_6_-related electronic polarons in riebeckite (at ~ 500 K) perfectly matches the increase in anisotropic conductivity with σ_||_ > σ_⊥_; (2) electron transport *via* polaron hopping is the dominant mechanism of charge transfer between 500 and 720 K; (3) anisotropy in the conductivity is strongly enhanced by increasing temperature (at 720 K σ_||_ ~ 4.7σ_⊥_); (4) conductivity data from MT measurements are best modelled by considering the effect of stress-driven alignment of amphibole crystals during plate motion.

Our work definitively demonstrates that the CPO of amphiboles, which is assumed to be among the main sources of seismic anisotropy observed worldwide in subduction zones, also strongly affects the electrical properties of subducting crustal rocks. This result emphasizes the further need for in situ HT Raman spectroscopy coupled with electrical-conductivity measurements on metamorphic rock-forming minerals to constrain the atomic-scale mechanisms of rock conductivity.

## Experimental methods

The sample used for the experiments described in this work is a near-end-member riebeckite from Mt. Malosa, Zomba District, Malawi. It has been comprehensively characterized in previous papers by a combination of analytical methods, including electron probe microanalysis, X-ray diffraction, Mössbauer spectroscopy, Fourier transform infrared (FTIR) spectroscopy, Raman spectroscopy, and X-ray absorption spectroscopy^35,36,42,62,63^. It has monoclinic *C*2/*m* symmetry at ambient conditions and its exact crystal-chemical formula is: ^A^(☐_0.9_K_0.06_Na_0.04_)^B^(Na_1.82_Ca_0.13_$\:{\text{F}\text{e}}_{0.05}^{2+}$)^C^[^*M*(1)^($\:{\text{F}\text{e}}_{1.84}^{2+}{\text{M}\text{g}}_{0.16}^{}$)^*M*(2)^($\:{\text{F}\text{e}}_{0.21}^{2+}{\text{M}\text{g}}_{0.04}^{}{\text{F}\text{e}}_{1.64}^{3+}{\text{A}\text{l}}_{0.10}^{}{\text{T}\text{i}}_{0.01}^{}$)^*M*(3)^($\:{\text{F}\text{e}}_{0.89}^{2+}{\text{M}\text{g}}_{0.06}^{}{\text{M}\text{n}}_{0.05}^{2+}$)]^T^(Si_7.97_Al_0.03_)O_22_^W^[(OH)_1.90_$\:{\text{F}}_{0.10}^{}$], that simplifies in the ^A^☐^B^Na_2_^C^(Fe^2+^_3_Fe^3+^_2_Si_8_O_22_(OH)_2_ stoichiometry.

Pristine fragments of a euhedral crystal (size ~ 1.5 × 1.5 × 2.5 mm^3^) were used for in situ high-temperature experiments under an external direct-current (dc) electric field applied with a Keithley 2410 high-voltage source meter (voltage basic accuracy of 0.012% and a low-current accuracy of 300 pA). Electrical contacts were glued to natural faces parallel either to the crystallographic (001) or (110) plane of an amphibole crystal using a PELCO high-performance silver paste and then annealed at 93 °C for 2 h. Hence, the applied dc electric field was either parallel (**E**_||_) or perpendicular (**E**_⊥_) to the crystallographic **c** axis, i.e., to the ribbons of FeO_6_ octahedra (see Fig. 2a and b). The area of the electrical contacts was measured under an optical microscope by moving the sample along the XYZ coordinates using a computer-controlled motorized stage. The structural stability of the sample was monitored throughout the experiment by examining the evolution of features in the Raman spectra. The conductivity of a specimen of commercial amorphous silica (Suprasil) with a size similar to that of the amphibole samples was used as a reference insulator to establish the “zero”-conductivity level for the experimental set-up. In situ temperature-dependent experiments were done in air using a LINKAM THMS-E600 stage with a temperature accuracy of 0.1 K and a heating rate of 10 K/min. Previous experiments^64^ on wide-band insulators using the same set-up verified the absence of ionic surface leakage up to 800 K. The stabilization time prior to collecting Raman spectra at the desired temperature was 10 min.

Raman spectra were collected with a Horiba Jobin-Yvon T64000 triple-monochromator spectrometer equipped with an Olympus BX41 microscope and a liquid-N_2_-cooled charge-couple-device detector, using the 514.532-nm line of a Coherent Innova 90 C FreD Ar^+^ laser. The spectrometer was calibrated to the Raman peak of Si at 520.5 cm^− 1^. The spectral resolution was ~ 2 cm^− 1^, whereas the instrumental precision in determining the peak positions was ~ 0.35 cm^− 1^.

Parallel polarized spectra (polarization of the incident light parallel to the polarization of scattered light) were collected in backscattering geometry with the **c**-axis parallel to the polarization of the incident light ($\:\stackrel{-}{y}\left(zz\right)y$ scattering geometry in Porto’s notation, with *z* || **c** and *y* ⊥ **c** and lying in the plane of **b-** and reciprocal-space a*-axes). The as-measured spectra were baseline-corrected, temperature-reduced for the Bose-Einstein population factor, and fitted with pseudo-Voigt functions to derive the phonon wavenumbers *ω*, full widths at a half maximum, integrated intensities *I*, and weight coefficients *q* of the Lorentzian contribution to the peak shape.

## Data Availability

The datasets generated and analysed during the current study are available from the corresponding authors on reasonable request.
